# Silent destruction: hidden muscle wasting and body decline in early Huntington’s disease

**DOI:** 10.1007/s00702-025-02991-3

**Published:** 2025-07-28

**Authors:** Marina Peball, Pia Schörghuber, Federico Carbone, Anne Zinganell, Franziska Di Pauli, Katarína Schwarzová, Atbin Djamshidian, Klaus Seppi, Beatrice Heim

**Affiliations:** 1 Department of Neurology, Medical University of Innsbruck, Anichstraße 35, 6020 Innsbruck, Austria; 2 Department of Neurology, District Hospital Kufstein, Endach 27, Kufstein, Austria

**Keywords:** Sarcopenia, Huntington’s disease, Frailty, Adipose tissue, Malnutrition

## Abstract

Huntington´s Disease (HD) is an autosomal dominant, neurodegenerative disorder with characteristic motor, behavioural, and cognitive impairment. Mutant Huntingtin may also affect peripheral tissue. We aimed to assess skeletal muscle (SMM) and fat mass, sarcopenia (EWGSOP2), and malnutrition in HD patients in early disease stages compared to age- and sex-matched healthy subjects. Body composition was evaluated by bioelectrical impedance analysis. The Unified HD Rating Scale and cognitive assessments were used for clinical characterization. Twenty early-stage HD patients (45% females) with a median age of 57 years, body mass index of 22 kg/m^2^, Total Motor Score of 17 points, and Total Functional Capacity of 10 points were included consecutively and prospectively. Confirmed sarcopenia (15%, n = 3) was uncommon. Appendicular SMM index was reduced in 60% (n = 12) and body fat mass in 35% (n = 7). SMM reduction was significantly associated with low weight (p = 0.049) and body fat (p = 0.048). Patients in disease-stage2 (n = 11) had a lower weight (p = 0.009) and body fat (p = 0.008) than patients in disease-stage1 (n = 9; i.e., patients without functional decline). Weight was also lower (p = 0.011) when compared to 20 healthy controls (45% females; median age 56 years). Fifty-five % of HD patients were at risk for malnutrition or malnourished (Mini Nutritional Assessment). The latter correlated with weight (r_s_ = 0.724, p < 0.001), SMM (r_s_ = 0.473, p = 0.035), body fat mass (r_s_ = 0.611, p = 0.004), motor symptoms (r_s_ = − 0.519, p = 0.019), independence (r_s_ = 0.450, p = 0.046), and executive function (r_s_ = 0.526, p = 0.017). Reduction of muscle or fat mass and malnutrition are common even in early-stage HD, which may contribute to progressive wasting and dependence.

## Introduction

Huntington´s Disease (HD) is an incurable neurodegenerative disorder caused by autosomal dominantly inherited elongation of cytosine-adenine-guanine (CAG) repeats in the Huntington gene on chromosome 4p16.3. Patients typically present with motor symptoms including chorea, dystonia, and hypokinetic features (e.g., bradykinesia, rigidity) as well as behavioural and psychiatric abnormalities. Progression of motor symptoms ultimately renders patients immobile causing functional dependency and frailty in HD. In clinical practice, HD is thus stratified into different disease stages based on motor, functional, as well as cognitive abilities (McColgan and Tabrizi 2018).

In addition to the characteristic neurological manifestations, HD patients may experience pathological changes in peripheral tissues including cardiomyopathy and skeletal muscle involvement (Carroll et al. 2015; Marchioretti et al. 2022; Chuang and Demontis 2021). Muscle pathology in HD may result from the toxic effects of mutant huntingtin (mHTT) or its aggregates, with mitochondrial dysfunction playing a key role in altered muscular energy metabolism observed even in presymptomatic mutation carriers (Ciammola et al. 2006, 2011; Zielonka et al. 2014; Saft et al. 2005). Evidence from clinical studies, cell cultures, and HD mouse models (notably R6/2 mice) indicates impaired mitochondrial depolarization, disrupted apoptotic signaling (e.g., cytochrome C release, elevated caspase-3, -8, and -9 levels), and reduced expression of metabolic regulators such as Peroxisome proliferator-activated receptor gamma coactivator 1 alpha (PGC-1α) (Ciammola et al. 2006; Chaturvedi et al. 2009). Mouse models demonstrate muscle atrophy, strength loss, fiber-type switching (from fast to slow-twich fibres), and neuromuscular junction abnormalities (Zielonka et al. 2014). Additionally, HD is associated with altered adipose tissue characterized by progressively reduced (truncal) fat mass (Costa de Miranda et al. 2019; Lakra et al. 2019). This is likely driven by mHTT-mediated apoptosis and dysregulation of fat storage and adipocyte differentiation via PGC-1α interference (Lakra et al. 2019; Phan et al. 2009).

From a clinical and patient-centred perspective, the progressive loss of skeletal muscle mass and function further compromises physical performance and mobility in HD. In addition, the reduction of peripheral muscle and adipose tissue may contribute to the development of a systemic body wasting syndrome, commonly referred to as progressive cachexia. This syndrome has been associated with increased mortality in HD mouse models and may similarly affect disease progression and prognosis in human patients (Chuang and Demontis 2021).

While both skeletal muscle degeneration and cachexia are predominantly observed in advanced stages of HD, the extent to which these alterations are present in earlier disease stages remains unclear. Therefore, in the present study, we aimed to systematically assess body weight, skeletal muscle mass, and body fat mass in patients with early-stage HD.

To evaluate muscle loss, we applied the established diagnostic criteria for sarcopenia, as defined by the European Working Group on Sarcopenia in Older People (EWGSOP). Sarcopenia summarizes muscle morbidity deriving from progressive loss of muscle strength, mass, and function. The 2019 diagnostic criteria from the EWGSOP (EWGSOP2) use a stepwise assessment approach with low muscle strength as the most important feature (i.e., probable sarcopenia). Additionally low muscle mass confirms sarcopenia (i.e., definite sarcopenia) and impaired physical function (e.g., low gait speed) classifies it as severe (Cruz-Jentoft et al. 2019). Primary sarcopenia, defined as age-dependent muscle impairment in otherwise healthy individuals, is prominent in the elderly population (11%; range 3 – 26% using EWGSOP2 criteria) (Fernandes et al. 2022). Muscle biology is further impaired in the presence of a chronic illness including neurodegenerative disorders such as HD, resulting in an increasing prevalence of sarcopenia (i.e., secondary sarcopenia) (Cruz-Jentoft et al. 2019). A nutritional assessment to evaluate malnourishment was integrated as an important extrinsic influential factor of body composition. Moreover, differences in body composition of HD patients and an age- and sex-matched healthy control group with adequate nutrition were evaluated.

We hypothesized that individuals in earlier disease stages despite preserved or only mildly impaired functional independence in daily activities, would already exhibit signs of secondary sarcopenia, characterized by reduced muscle mass and function.

Furthermore, we postulated that a concurrent reduction in body fat mass may indicate the early onset of a systemic body wasting syndrome. We additionally expected that HD patients would demonstrate significantly lower muscle mass, body weight, and fat mass compared to age- and sex-matched healthy controls. Finally, we anticipated that incorporating a structured assessment of nutritional status would offer valuable insights into potentially modifiable risk factors contributing to cachexia in HD.

## Methods

### Study participants

The study was approved by the local ethics committee. All individuals gave written informed consent before participation. No participant received a stipend. All procedures were performed in accordance with the 1964 Helsinki declaration and its later amendments.

We consecutively recruited HD patients with genetically confirmed HD and typical motor symptoms of the disease (diagnostic confidence level = 4; Unified HD rating scale [UHDRS] Total Motor Score [TMS] > 5). Moreover, we recruited an age- and sex-matched healthy control group for comparison of the body composition parameters. No family controls were used to avoid confounders (such as similar physical exercise routines or meals or yet undiagnosed HD mutation carrier status).

Clinical assessment of HD patients included the UHDRS TMS for motor symptoms, UHDRS Total Functional Capacity (TFC) for functionality in daily living, UHDRS Independence Score (IS) for dependency in daily activities, the Symbol Digit Modalities Test (SDMT) for psychomotor speed, and the Mini Mental State Exam (MMSE) for global cognition (Mestre et al. 2018; Abreu et al. 2021).

Patients were categorized into clinical stages according to their functional capacities (UHDRS total functional capacity, UHDRS-TFC): stage 1: 11 ≤ UHDRS-TFC ≤ 13; stage 2: 7 ≤ UHDRS-TFC ≤ 10; stage 3: 3 ≤ UHDRS-TFC ≤ 6; stage 4: 1 ≤ UHDRS-TFC ≤ 2 (Shoulson and Fahn 1979). We did not include patients with disease stages 3 or 4, representing advanced HD disease stages, as our hypotheses specifically focused on early-stage pathology prior to the onset of profound functional decline and systemic wasting.

The CAG-Age-Product (CAP) score estimates the disease state of a patient, reflecting the exposure to the toxic effects of the mutant HTT protein. The CAP score is calculated as age x (CAG – 33.66) (Zhang et al. 2011). A score of 100 indicates that the patient has reached the expected age at onset (AAO) of motor symptoms.

### Assessment of sarcopenia, body composition and nutrition

The diagnosis of sarcopenia was based on the EWGSOP´s operating standards and sex-specific cut-offs (Cruz-Jentoft et al. 2019). Muscle mass was measured with the InBody 770 bioelectrical impedance analysis (BIA) machine at 50 kHz (InBody Co., Ltd, Seoul, Korea). Appendicular skeletal muscle mass (ASM) was calculated based on the recommended equation by Sergi et al. (2015). ASM was normalized with height (ASM Index, ASMI, in kg/m^2^). For defining reduced ASMI, the sex-specific cut-off values of the EWGSOP2 were used (Cruz-Jentoft et al. 2019). Muscle strength was assessed with a calibrated handheld dynamometer (CITEC CT3002). Two trials for each hand were performed and the best result from the strongest hand was used. To examine muscle function, we measured gait speed (in meters/second) on a marked track with a stopwatch.

We also assessed the usability of the SARC-F as a screening questionnaire for sarcopenia in HD patients. The SARC-F is a simple questionnaire composed of five items: strength, climbing stairs, assistance with walking, rising from a chair and falls with zero to two points each. A value of ≥ four points is indicative of sarcopenia (Malmstrom et al. 2016).

To assess vulnerability and an increased risk for negative health outcomes, we evaluated the items of the physical frailty phenotype in our HD patient cohort, which is defined as the presence of ≥ three of the following: unintentional weight loss (> 10 pounds during the last year), exhaustion (> 1 point on question 7 or 20 of the Center for Epidemiologic Studies-Depression Scale), low physical activity (< 383 kcal peer week for males and < 270 kcal for females), low gait speed (sex- and height-dependent), or low grip strength (sex- and Body Mass Index-dependent) (Fried et al. 2001). A screening for frailty was performed using the 9-item screening tool Canadian Study of Health and Aging Clinical Frailty Scale (CSHA CFS, Version 1). The latter is based on impairment in mobility, functional capacity, cognition, and comorbidities with a score of ≥ four points being indicative of frailty (Rockwood and Theou 2020).

Apart from muscle mass, BIA was used to assess weight (in kg), body fat mass (BFM, in kg and %), visceral fat area (VFA, in cm2), and fat-free mass (FFM, i.e., body mass that is not adipose tissue; kg). Individual sex- and height-dependant reference values as provided by InBody were used (“normal, i.e., within the sex- and height-dependant reference range; “reduced”; “increased”), except for VFA (cut-off > 100 cm^2^). BIA also provided 50 kHz whole body phase angle (in °).

The Mini Nutritional Assessment (MNA) is a validated tool to assess nutritional status. It was originally developed to assess malnutrition in the elderly (Guigoz et al. 2002). However, it has been widely used in younger populations and has shown good predictive properties for negative health outcomes (e.g., hospital readmission, death) (Pereira Bertini de Oliveira et al. 2023; Aggarwal et al. 2013). In this study, we used the long form (screening and assessment) of the German version of the MNA with 24 to 30 points representing a normal nutritional status, 17 to 23.5 points representing a person at risk for malnutrition, and a score of < 17 points indicating malnutrition (Guigoz et al. 2002). We did not instrumentally assess dysphagia in our cohort. Calf circumference was obtained as part of the anthropometric measures of the MNA measuring the widest part of the calf while having the legs hang loosely in the sitting position. For the MNA, the measurement of the left leg was used as specified in the instructions. For other statistical analysis, the mean calf circumference of both legs was used in this study. Based on the specifications of the MNA, a cut-off of < 31 cm was classified as low calf circumference.

Exclusion criteria for participation were presence of another movement disorder or any contraindications to perform BIA (e.g., pacemaker).

### Statistical analyses

Categorical variables are given in number (n) and percent (%) of the category (sex, unintentional weight loss in the past year, MNA result, number of patients with low calf circumference, number of patients with positive screening for sarcopenia and frailty, number of patients with no/probable/confirmed/severe sarcopenia and no/pre-/ and frailty, number of patients with normal/reduced/increased body composition parameters). For continuous quantitative measures the mean with its standard deviation and the median were calculated. Group comparisons were made between HD patients and healthy controls as well as different disease stages (stage 1 vs. stage 2). Continuous variables were tested for normality with the Kolmogorov–Smirnov test and Mann–Whitney-U tests, unpaired student´s t-tests or Analysis of Variance (ANOVA) were used for group comparisons depending on the scale type (see Table legends for details). Spearman's rank-order correlation (r_s_) was used to test for any relationship of MNA score (not-normally distributed) with clinical, and body composition parameters of HD patients. Correlation coefficient (r_s_) absolute values between 0 and 0.09 are a negligible correlation, 0.1 to 0.39 a weak correlation, 0.4 to 0.69 a moderate correlation, 0.7 to 0.89 a strong correlation, and 0.9 to 1.0 a very strong correlation (Schober et al. 2018). The significance level was set at a two-sided p-value of < 0.05. SPSS 25.0 for windows (SPSS Inc., IBM Corporation and other(s) 1989, 2013, Chicago, IL) was used to tabulate and analyse data.

## Results

Demographic and clinical data of the 20 included HD patients are summarised in Table 1. The screening for sarcopenia was positive in 25% (n = 5) and for frailty in 15% (n = 3). Only 15% (n = 3) classified as sarcopenic and 10% (n = 2) as frail according to EWGSOP2 and the physical frailty phenotype, respectively. Handgrip strength was below the sex-specific cut-off provided by the EWGSOP2 in 9 patients (45%). Gait speed (per EGWSOP2 criteria) and calf circumference (cut-off < 31 cm) were not reduced in any patient. The ASMI was reduced in 60% (n = 12, EWGSOP2 cut-off). Fifty-five % of patients had a normal individual weight (n = 11), 20% (n = 4) low and 25% (n = 5) high weight. Unintentional weight loss was present in 25% (n = 5). BFM (in kg) was each normal and reduced in 35% (n = 7), but high in 30% (n = 6, individual cut-off) The VFA was increased in 25% (n = 5) of the patient cohort (Table 1). The MNA revealed 50% (n = 10) of patients to be at risk of malnutrition and 5% (n = 1) to be malnourished. Forty-five % (n = 9) had a normal nutritional status (Table 1).

**Table 1:** Demographic and clinical characteristics as well as body composition parameters of the Huntington's disease patient cohort

	Total (n = 20)	Stage 1 (n = 9)	Stage 2 (n = 11)	p-value
Age (in years)	53.49 ± 9.97 (56.46)	49.91 ± 8.98 (53.50)	56.43 ± 10.16 (60.42)	0.138
Sex (female/male)	9 (45%)/11 (55%)	4 (44.4%)/5 (55.6%)	5 (45.5%)/6 (54.5%)	1.000
CAG repeat length on the longer allele	44.10 ± 3.06 (44.00)	43.00 ± 2.18 (42.00)	45.00 ± 3.46 (44.00)	0.134
Height (in m)	1.73 ± 0.09 (1.72)	1.74 ± 0.09 (1.79)	1.72 ± 0.09 (1.72)	0.517
Weight (in kg)^a^	71.73 ± 18.95 (68.20)	80.47 ± 23.03 (79.90)	64.57 ± 11.55 (62.20)	0.009
Normal individual weight	11 (55%)	4 (44.4%)	7 (63.6%)	0.178
Low weight	4 (20%)	1 (11.1%)	3 (27.3%)	
High weight	5 (25%)	4 (44.4%)	1 (9.1%)	
Unintentional weight loss in the last year	5 (25%)	0 (0%)	5 (45.5%)	0.317
BMI (in kg/m^2^)	23.88 ± 5.73 (22.14)	26.32 ± 7.00 (24.12)	21.88 ± 3.66 (21.22)	0.013
UHDRS TMS	18.10 ± 9.38 (17.00)	12.44 ± 7.86 (16.00)	22.73 ± 8.08 (21.00)	0.010
SDMT	27.30 ± 11.29 (23.50)	35.44 ± 11.05 (33.00)	20.64 ± 6.01 (22.00)	0.004
UHDRS TFC	10.55 ± 2.04 (10.00)	12.33 ± 1.00 (13.00)	9.09 ± 1.38 (10.00)	< 0.001
UHDRS IS (in %)	88.50 ± 11.82 (90.00)	96.67 ± 5.00 (100.00)	81.82 ± 11.68 (90.00)	0.001
MMSE	27.45 ± 2.01 (28.00)	28.67 ± 1.23 (29.00)	26.45 ± 2.02 (27.00)	0.008
MNA	23.03 ± 3.83 (23.50)	24.00 ± 3.66 (25.50)	22.23 ± 3.95 (23.50)	0.165
Normal nutritional status	9 (45%)	5 (55.6%)	4 (36.4%)	0.516
At risk of malnutrition	10 (50%)	4 (44.4%)	6 (54.5%)	
Malnourished	1 (5%)	0	1 (9.1%)	
Calf circumference (cm)	37.16 ± 4.17 (36.00)	39.00 ± 5.24 (39.25)	35.66 ± 2.37 (35.00)	0.021
Calf circumference < 31 cm	0	0	0	NA
SARC-F score	1.75 ± 1.83 (1.50)	1.22 ± 1.99 (0.00)	2.18 ± 1.66 (2.00)	0.265
Sarcopenia screening positive (≥ 4 pts.)	15 (25%)	2 (22.2%)	3 (27.3%)	1.000
CSHA CFS	2.55 ± 0.89 (2.50)	2.67 ± 1.00 (3.00)	2.45 ± 0.82 (2.00)	0.617
Frailty screening positive (≥ 4 pts.)	3 (15%)	2 (22.2%)	1 (9.1%)	0.566
Physical frailty phenotype				
Not frail	3 (15%)	1 (11.1%)	2 (18.2%)	0.904
Prefrail	15 (75%)	7 (77.8%)	8 (72.7%)	
Frail	2 (10%)	1 (11.1%)	1 (9.1%)	
Sarcopenia (EWGSOP2)				
No sarcopenia	11 (55%)	5 (55.6%)	6 (54.5%)	0.166
Probable sarcopenia	6 (30%)	4 (44.4%)	2 (18.2%)	
Confirmed Sarcopenia	3 (15%)	0	3 (27.3%)	
Severe Sarcopenia	0	0	0	
ASMI (in kg/m^2^)	6.23 ± 0.99 (6.16)	6.53 ± 1.21 (6.47)	5.98 ± 0.74 (6.05)	0.250
Reduced^b^	12 (60%)	4 (44.4%)	8 (72.7%)	0.362
Handgrip strength, right side (in kg)	23.36 ± 8.68 (22.84)	22.71 ± 11.58 (23.64)	23.89 ± 5.93 (21.62)	0.787
Handgrip strength, left side (in kg)	20.82 ± 9.44 (21.11)	22.55 ± 12.45 (21.83)	19.40 ± 6.35 (20.60)	0.504
Gait velocity (in m/sec)	1.39 ± 0.42 (1.26)	1.36 ± 0.26 (1.33)	1.41 ± 0.53 (1.18)	0.810
Body Fat Mass (in kg)^a^	19.00 ± 14.12 (14.95)	21.89 ± 15.80 (15.00)	16.64 ± 12.87 (13.90)	0.008
Normal Reduced Increased	7 (35%) 7 (35%) 6 (30%)	2 (22.2%) 3 (33.3%) 4 (44.4%)	5 (45.5%) 4 (36.4%) 2 (18.2%)	0.384
Body Fat Mass (in %)^a^	22.86 ± 12.03 (19.90)	22.57 ± 16.01 (18.80)	20.96 ± 10.65 (20.20)	0.312
n of patients with normal % of body fat mass	9 (45%)	4 (44.4%)	5 (45.5%)	0.587
Reduced % of body fat mass	4 (20%)	1 (11.1%)	3 (27.3%)	
Increased % of body fat Mass	7 (35%)	4 (44.4%)	3 (27.3%)	
Visceral Fat area (in cm^2^)	86.39 ± 65.78 (71.55)	108.04 ± 81.88 (72.50)	68.66 ± 45.72 (70.40)	0.043
Increased (> 100 cm^2^)	5 (25%)	3 (33.3%)	2 (18.2%)	0.617
Fat-free mass (in kg)^a^	54.18 ± 12.77 (50.90)	58.58 ± 15.43 (58.10)	50.57 ± 9.37 (48.40)	0.008
Normal	15 (75%)	7 (77.8%)	8 (72.7%)	0.086
Reduced	3 (15%)	0	3 (27.3%)	
Increased	2 (10%)	2 (22.2%)	0	
50 kHz Whole Body Phase angle (in °)	5.08 ± 0.78 (5.15)	5.39 ± 0.90 (5.20)	4.83 ± 0.58 (4.50)	0.093

Continuous data is presented as mean ± SD (median), categorical data as n, %. Significance level: two-sided p ≤ 0.05. Unpaired T-test, ANOVA or Mann–Whitney-U test for continuous variables, Qui-Square test or Fisher´s exact test for nominal variables (depending on the group size). Group comparisons were based on disease stages

*CAG* cytosine-adenine-guanine, *BMI* Body Mass Index, *UHDRS* Unified Huntington’s Disease Rating Scale, *TMS* Total Motor Score, *SDMT* Symbol-Digit-Modalities-Test, *TFC* Total Functional Capacity, *IS* Independence Score, *MMSE* Mini Mental State Exam. *MNA* Mini Nutritional Assessment, *SARC-F* a simple five‐item questionnaire for Sarcopenia, *CSHA* Canadian Study on Health & Aging, *CFS* Clinical Frailty Scale, *EWGSOP* European Working Group on Sarcopenia in Older People, *ASMI* Appendicular skeletal muscle mass index (= Appendicular skeletal muscle mass/height in metre^2^), *NA* not applicable, *n* number

^a^Reference values as provided by InBody (personalized minimum and maximum ideal measurement) were used

^b^Reference values of the EWGSOP 2019 criteria for muscle mass loss in sarcopenia were used

We included 9 patients in disease stage 1 (45%, four females, five males) and 11 patients in stage 2 (55%, five females, six males). CAP score results were congruent with disease staging. All 3 confirmed sarcopenia cases (15%) had a disease stage of 2. The overall prevalences of sarcopenia and its single components (ASMI, grip strength, gait velocity) were not different between disease stages 1 and 2 (all p > 0.250, Table 1). Compared to disease stage 1, patients in stage 2 had a significantly decreased calf circumference (p = 0.021), body weight (p = 0.009), BFM (p = 0.008 in kg), and VFA (p = 0.043). The percentage of BFM (p = 0.312) and the MNA (p = 0.165) were not statistically different between disease stage 1 and 2 (Table 1). Regarding clinical characteristics besides UHDRS TMS (p = 0.010), TFC, and IS, patients in stage 2 had a lower SDMT score (p = 0.004) and MMSE (p = 0.008, Table 1).

Table 2 displays characteristics of patients stratified by ASMI. Low ASMI was significantly associated with low body weight (p = 0.049), BMI (p = 0.023), body fat (p = 0.048 in kg, p = 0.019 in %), and VFA (p = 0.007). Neither the MNA, UHDRS TMS, TFC, IS, SDMT nor frailty were significantly different between the groups (all p > 0.082).

**Table 2:** Associations of reduced appendicular skeletal muscle mass index with relevant body composition parameters

	Patients with low ASMI (n = 12)^a^	Patients with normal ASMI (n = 8)^a^	p-value
Weight (in kg)	65.02 ± 13.21 (63.70)	81.79 ± 22.54 (78.90)	0.049
BMI (in kg/m^2^)	21.16 ± 3.00 (20.83)	27.96 ± 6.57 (27.45)	0.023
UHDRS TMS	19.92 ± 9.86 (21.00)	15.38 ± 8.47 (16.50)	0.287
UHDRS TFC	10.17 ± 1.90 (10.00)	11.13 ± 2.23 (12.00)	0.336
UHDRS IS (in %)	87.50 ± 11.38 (90.00)	90.00 ± 13.09 (95.00)	0.656
SDMT	26.50 ± 12.00 (25.00)	28.50 ± 10.82 (23.00)	0.709
Physical Frailty phenotype			
Not frail	3 (25%)	0 (0%)	0.082
Prefrail	9 (75%)	6 (75%)	
Frail	0 (0%)	2 (25%)	
MNA	22.63 ± 3.74 (23.00)	23.63 ± 4.14 (24.75)	0.581
Body Fat Mass (in kg)	13.98 ± 11.88 (9.20)	26.54 ± 14.54 (25.85)	0.048
Body Fat Mass (in %)	17.23 ± 7.56 (16.55)	31.31 ± 12.92 (25.90)	0.019
Visceral Fat Area (in cm^2^)	53.83 ± 29.81 (39.90)	135.23 ± 76.20 (123.65)	0.007
50 kHz Whole Body Phase angle (in °)	5.02 ± 0.64 (5.20)	5.18 ± 0.99 (5.00)	0.667

Continuous data is presented as mean ± SD (median), categorical data as n, %. Significance level: two-sided p ≤ 0.05. Unpaired T-test or Mann–Whitney-U test for continuous variables

*BMI* Body Mass Index, *UHDRS* Unified Huntington’s Disease Rating Scale, *TMS* Total Motor Score, *SDMT* Symbol-Digit-Modalities-Test, *TFC* Total Functional Capacity, *IS* Independence Score, *MNA* Mini Nutritional Assessment, *ASMI* Appendicular skeletal muscle mass index (= Appendicular skeletal muscle mass / height in metre^2^), *n* number

^a^Reference values of the EWGSOP 2019 criteria for muscle mass loss in sarcopenia were used

Table 3 represents relevant correlations of the MNA. The MNA strongly positively correlated with weight (r_s_ = 0.724, p < 0.001) and BMI (r_s_ = 0.741, p < 0.001). Moderate positive correlations were found for MNA and ASMI (r_s_ = 0.473, p = 0.035), FFM (r_s_ = 0.468, p = 0.037), BFM in kg (r_s_ = 0.611, p = 0.004), VFA (r_s_ = 0.565, p = 0.009), SDMT (r_s_ = 0.526, p = 0.017), and UHDRS IS (r_s_ = 0.450, p = 0.046). A moderate inverse correlation existed with the UHDRS TMS (r_s_ = -0.519, p = 0.019). No correlation was found with sarcopenia (EWGSOP2), frailty, and the UHDRS TFC (all p > 0.101).

**Table 3:** Correlations for the mini nutritional assessment with sarcopenia, frailty, relevant body composition parameters and clinical characteristics

Spearman correlation of the MNA with	Correlation coefficient r_s_	p-value
Weight (in kg)	0.724	< 0.001
BMI (in kg/m^2^)	0.741	< 0.001
UHDRS TMS	− 0.519	0.019
UHDRS TFC	0.377	0.101
UHDRS IS (in %)	0.450	0.046
SDMT	0.526	0.017
Physical Frailty phenotype	− 0.256	0.276
Sarcopenia	− 0.192	0.417
ASMI (in kg/m^2^)	0.473	0.035
Body Fat Mass (in kg)	0.611	0.004
Body Fat Mass (in %)	0.421	0.064
Visceral Fat Area (in cm^2^)	0.565	0.009
Fat Free Mass (in kg)	0.468	0.037
50 kHz Whole Body Phase angle (in °)	0.560	0.010

Spearman's rank-order correlation (r_s_) of 0.1, 0.3, and 0.5 were considered ‘small’, ‘medium’, and ‘large’ effect

*BMI* Body Mass Index, *UHDRS* Unified Huntington’s Disease Rating Scale, *TMS* Total Motor Score, *TFC* Total Functional Capacity, *IS* Independence Score, *SDMT* Symbol-Digit-Modalities-Test, *ASMI* Appendicular skeletal muscle mass index (= Appendicular skeletal muscle mass/height in metre^2^)

Table 4 compares the body composition parameters of our HD patient cohort to an age- (p = 0.809) and sex-matched (p = 1.000) healthy control group (n = 20). The healthy control group did not differ significantly in height, weight, BMI, ASMI, BFM, VFA, and FFM compared to the total HD patient group (all p > 0.303) and to HD patients in disease stage 1 (all p > 0.412). HD patients in disease stage 2 had a lower weight (p = 0.011) and BMI (p = 0.041). The ASMI showed a tendency to be reduced in HD patients of disease stage 2 (median: 5.99 kg/m^2^) compared to healthy controls (median: 6.47 kg/m^2^, p = 0.073 and p = 0.081) while body fat mass and visceral fat area were not statistically different (all p > 0.189, Table 4).

**Table 4:** Demographic characteristics and body composition parameters of matched healthy controls

	Healthy controls total (n = 20)	p-value (compared to Total HD Group, n = 20)	p-value (compared to stage 1 HD patient group, n = 9)	p-value (compared to stage 2 HD patient group, n = 11)
Age (in years)	52.76 ± 9.14 (55.71)	0.809	0.443	0.331
Sex (female/male)	9 (45%)/11 (55%)	1.000	1.000	1.000
Height (in m)	1.75 ± 0.09 (1.76)	0.345	0.730	0.262
Weight (in kg)^a^	77.00 ± 12.63 (76.20)	0.307	0.680	0.011
Normal individual weight Low weight High weight	8 (40%) 1 (5%) 11 (55%)	0.104	0.779	0.025
BMI (in kg/m^2^)	25.00 ± 4.13 (26.10)	0.479	0.612	0.041
ASMI (in kg/m^2^)	6.54 ± 0.91 (6.47)	0.303	0.986	0.073
Reduced^b^	8 (40%)	0.206	1.000	0.081
Body Fat Mass (in kg)^a^	19.13 ± 8.78 (17.70)	0.972	0.549	0.575
Normal	7 (35%)	0.492	0.676	0.305
Reduced	4 (20%)			
Increased	9 (45%)			
Body Fat Mass (in %)^a^	24.47 ± 9.64 (22.35)	0.643	0.892	0.376
n of patients with normal % of Body Fat Mass	10 (50%)	0.349	0.829	0.189
Reduced % of Body Fat Mass	1 (5%)			
Increased % of Body Fat Mass	9 (45%)			
Visceral Fat area (in cm^2^)	88.54 ± 44.85 (77.70)	0.904	0.412	0.257
Increased (> 100 cm^2^)	5 (25%)	1.000	0.675	1.000
Fat-free mass (in kg)^a^	57.87 ± 10.41 (58.75)	0.323	0.902	0.059
Normal	11 (55%)	0.166	0.418	0.063
Reduced	2 (10%)			
Increased	7 (35%)			
50 kHz Whole Body Phase angle (in °)	5.58 ± 0.68 (5.56)	0.038	0.587	0.004

Continuous data is presented as mean ± SD (median), categorical data as n, %. P-value for comparison to the Huntington´s disease patient cohort (Total/stage 2/stage 3). Significance level: two-sided p ≤ 0.05. Unpaired T-test for continuous variables (all normally distributed), Qui-Square test or Fisher´s exact test for nominal variables (depending on the group size)

*BMI* Body Mass Index, *ASMI* Appendicular skeletal muscle mass index (= Appendicular skeletal muscle mass/height in metre^2^), *HD* Huntington´s Disease, *n* number

^a^Reference values as provided by InBody (personalized minimum and maximum ideal measurement) were used

^b^Reference values of the EWGSOP 2019 criteria for muscle mass loss in sarcopenia were used

## Discussion

In this pilot study, 20 HD patients in early disease stages 1 or 2 were assessed for sarcopenia and its components, body weight and body fat mass, as well as the extrinsic influence of nutritional intake. Their body composition was compared to that of 20 age- and sex-matched healthy controls, both as a total group and stratified by disease stage.

Contrary to our initial hypothesis, confirmed secondary sarcopenia as per EWGSOP2 criteria was uncommon (15%, n = 3) and unrelated to disease stage in our HD patient cohort. Regarding its components, muscle function (gait velocity) was largely preserved. However, muscle strength (handheld dynamometer) was reduced in 45% (n = 9) and muscle mass per height (ASMI) was even reduced in 60% (n = 12) of our early-stage HD cohort. This was associated with reduced body weight and body fat mass but not with clinical disease scores (i.e. UHDRS TMS, TFC, IS, SDMT). Overall, body weight and fat mass declined progressively with advancing HD disease stage.

Compared to age- and sex-matched healthy controls, HD patients with disease stage 2 had a lower weight and BMI with comparable BFM. The ASMI was also lower in HD patients with disease stage 2 compared to control subjects, although not reaching statistical significance (p = 0.073).

Peripheral involvement in HD pathology is known from preclinical (e.g., Ciammola et al. 2006; Zielonka et al. 2014) as well as clinical research and mostly attributed to disruption of cellular mechanisms (e.g., energy metabolism) by neurodegeneration, dysfunctional mutant HTT-protein itself or its aggregates (Carroll et al. 2015; Turner et al. 2007). Muscle metabolism pathology is apparent not only in manifest HD patients but also in premanifest mutation carriers (Ciammola et al. 2011; Saft et al. 2005). A summary of data from preclinical studies was provided in the introduction. Data from clinical studies on muscle mass and function in HD however is limited and conflicting. Supporting our results, one study found reduced lean body mass (as assessed by dual-energy x-ray absorptiometry) in 21 HD patients compared to matched healthy control subjects (Costa de Miranda et al. 2019). Reduction of lower extremity muscle strength by an average of 50% was also seen in 20 HD patients vs. controls (Busse et al. 2008). Contrary to our results, other studies found unremarkable fat-free mass (measured by dual-energy x-ray absorptiometry or BIA) in HD patients, mostly compared to family-controls (Cubo et al. 2015; Pratley et al. 2000; Sussmuth et al. 2015). Fat-free mass reduction in these studies was not defined by cut-off values and the cohorts differ from our participants in disease characteristics (e.g., disease stages), thus limiting comparability. The similarity between body composition of HD patients and family members as controls hints towards confounders such as nutrition or physical exercise routine. The aforementioned clinical studies did not categorize muscle wasting according to standardized criteria for sarcopenia. To the best of our knowledge, no study so far assessed sarcopenia with all its components in HD patients, making this a unique pilot study. Standardized characterization of muscle wasting in HD is essential to ensure consistency across trial methods and reporting, to improve comparability between research findings, to enable early detection (e.g., in a preclinical stage) via the use of consistent diagnostic benchmarks, and to reliably assess muscle loss progression. It also facilitates the design of robust clinical trials and therapeutic strategies. This study represents an initial effort toward establishing a standardized characterization of muscle wasting in HD patients.

Muscle impairment in our study participants was associated with reduced body weight and fat mass. As with muscle mass, clinical data on body fat mass measurements in HD is lacking. One study found a reduction of truncal fat mass in HD patients compared to controls (Costa de Miranda et al. 2019). Despite the lack of data, the link between body fat mass, muscle mass, and body weight loss as a prominent feature in HD patients is recognised (e.g., Costa de Miranda et al. 2019; Chuang and Demontis 2021). Weight loss in HD is immanent to the disease itself, independent of motor features, and progresses with disease stages, which is reflected in our findings, especially when compared to healthy controls. Altered adipose tissue function and an increased energy expenditure contribute to weight loss in HD (Carroll et al. 2015; Aziz et al. 2010a, 2010b; Aziz et al. 2008). Unintentional loss of muscle, fat, and weight combined characterize cachexia, which may have consequences beyond functional impairment, as it is a well-known contributor to overall mortality. The European Society of Clinical Nutrition and Metabolism (ESPEN) recommends an early nutritional intervention in pre-cachexia as one important preventive measure for cachexia and its life-threatening sequelae (Biolo et al. 2014).

In our early-stage HD patient cohort, 55% were at risk for malnutrition or malnourished according to the MNA. Patients with an insufficient nutritional status had lower weight, BMI, ASMI, and BFM. Inadequate nutritional intake may therefore be a key factor for weight loss in HD. This result is alarming with respect to the concept of cachexia. Previous research in HD patients confirms the correlation of insufficient nutritional intake (e.g., caloric and proteins) to fat-free mass reduction (Sussmuth et al. 2015; Cubo et al. 2015). Malnutrition in HD may be a common pathway of different pathologies, including functional dependency, cognitive decline, motor impairment, and dysphagia. Supporting this, nutritional status in our study was worse in HD patients with higher functional dependency (IS), executive dysfunction (SDMT), and greater motor symptom burden (TMS), even when the overall UHDRS TMS was low. Motor difficulties may not only directly impair food intake but also limit the ability to shop for food or prepare meals (Trejo et al. 2004). Dysphagia has been associated with older age, higher UHDRS motor scores, poor cognitive status, dysphonia, dysarthria, and impaired tongue movements in patients with HD (Pizzorni et al. 2020). Although these features are more commonly observed in advanced disease stages, systematic swallowing assessments have demonstrated that dysphagia may already be prevalent in the early disease course (Sapmaz Atalar et al. 2024). Swallowing difficulties may result in reduced caloric intake and inefficient nutrient absorption, ultimately leading to weight loss (Gaba 2025). This may progress to cachexia, particularly when combined with the increased energy expenditure associated with HD and chorea. Despite its significant clinical impact, dysphagia in HD has received relatively limited research attention (Pizzorni et al. 2020). Given its potentially severe consequences as mentioned above but also including aspiration and related mortality, early and routine assessment is essential. Unfortunately, dysphagia is not captured by the UHDRS TMS. Clinical assessment tools (e.g. questionnaires) should be complemented by instrumental swallowing evaluation. Videofluoroscopic and endoscopic methods have been shown to be feasible in this population and should be integrated into comprehensive care and research protocols (Pizzorni et al. 2020).

The findings of our study provide evidence that malnutrition is a critical and underrecognized condition even in early-stage HD patients. By demonstrating a high prevalence of malnutrition and its association with impaired body composition and function, this study offers new insights into the role of inadequate nutritional intake as a modifiable, extrinsic contributor to body health in HD. This represents a significant step forward in understanding the multifactorial nature of cachexia in HD. Our study underscores the urgent need for routine nutritional screening and implementation of strategies to improve nutritional status and thus cachexia in HD patients. Monitoring of risk factors, modification of the food (e.g., texture, frequency of intake), assessment of caloric requirements, and optimal symptomatic control are key (Trejo et al. 2004). Adherence to a Mediterranean Diet for example has been proven beneficial in HD patients in previous studies (Christodoulou et al. 2020).

Besides nutritional interventions, counteractive measurements for muscle mass loss should include physical training. In the general population, endurance and resistance training are recommended in patients with (pre-)cachexia to improve skeletal muscle pathology (Biolo et al. 2014). In HD patients, physical activity had a positive impact on gait, balance, overall motor function, and quality of life (Trovato et al. 2022). A long-term multi-component training (group aerobic and resistance training as well as home-based exercise) in 20 early-to-middle-stage HD patients not only improved UHDRS TMS, chorea, and tandem walking, but also fat-free mass and muscle strength compared to a control group (Thompson et al. 2013). Physical exercise also has the potential to increase grey matter volume (e.g., caudate nucleus), learning, and memory in HD patients (Cruickshank et al. 2015). Pharmacological interventions to prevent muscle mass loss have yet to be established in HD patients.

Apart from implications for treatment, muscle pathology may also represent a peripheral biomarker for HD progression, which can be easily assessed e.g., by using BIA.

Our study has several strengths. The standardized assessment of muscle pathology according to the EWGSOP2 criteria as well as the integration of a nutritional assessment are important strengths of this study. We used BIA as a standardized and validated method (Cruz-Jentoft et al. 2019). ASM was calculated using raw BIA measurements at 50 kHz based on a recommended equation (Sergi et al. 2015). The equation itself was based on a European cohort and is therefore reflective of our patients. To reduce influence of body size and improve comparability, the ASM was adjusted (ASM/height^2^, i.e., ASMI). Considering these, the easy use, and wide availability of BIA, it is a good method in comparison to dual-energy X-ray absorptiometry, which has long been the gold standard for assessment of body composition (Cruz-Jentoft et al. 2019). Clinical evaluation of HD patients was performed prospectively by movement disorder specialists based on standard criteria. This guarantees the correct diagnosis and classification of disease characteristics and stages. Lastly, the body composition data was compared to an age- and sex-matched healthy control group without blood relation to the HD subjects. Nonetheless, there are also some limitations to consider. Firstly, this is a cross-sectional study design. Secondly, our aim was to include early-stage HD patients. As HD is already a rare disease, our approach further reduces the population of suitable patients. The sample size however is comparable to previous studies on this topic (e.g., Thompson et al. 2013; Busse et al. 2008). Nevertheless, it may limit generalizability. Lastly, we did not include an instrumental swallowing assessment as it was not the primary scope of our study, which limits our ability to evaluate the contribution of dysphagia to nutritional status. This would complement our malnutrition assessment that is based on questionnaire and anthropometric measurements.

## Conclusion

Beyond the well-established neurological features of HD, there is growing evidence of peripheral involvement in HD pathology. This study provides evidence of early peripheral involvement, including reductions in muscle mass, fat mass, and body weight — even in patients with preserved daily function. Weight loss progressed with disease stage and differed significantly from healthy controls. Moreover, malnutrition was found to be a common feature already in early-stage HD patients and was associated with motor and cognitive impairment, as well as functional dependency.

This is not only the first study to evaluate muscle pathology in a standardized way using the EWGSOP2 criteria for sarcopenia. It also provides a novel contribution to understanding the cachexia pathway in HD.

Our findings highlight the importance of preservation of body health and adequate nutrition in HD patients, rendering them important targets for intervention with a potential to improve disease burden. The optimal treatment strategy in this specific patient cohort must be assessed in future clinical trials as well as the impact of peripheral muscle and adipose tissue involvement on disease progression.

## Data Availability

Data supporting the findings of this study are available from the first or corresponding author upon reasonable request and fulfilling data sharing regulations approved by the local ethics committee. Only deidentified individual data that underlie the results reported in this manuscript will be made available. Proposals should be directed to the first or corresponding author. Data will be available beginning three months and ending five years following article publication solely for the purpose of achieving aims in the approved proposal. Data will only be shared via individual secured network connections.
